# Activity patterns are associated with fractional lifespan, memory, and gait speed in aged dogs

**DOI:** 10.1038/s41598-023-29181-z

**Published:** 2023-02-14

**Authors:** Alejandra Mondino, Michael Khan, Beth Case, Sara Giovagnoli, Andrea Thomson, B. Duncan X. Lascelles, Margaret Gruen, Natasha Olby

**Affiliations:** 1 Department of Clinical Sciences, College of Veterinary Medicine, North Carolina State University, Raleigh, NC USA; 2 Department of Psychology “Renzo Canestrari”, University of Bologna, Bologna, Italy; 3 Translational Research in Pain, Department of Clinical Sciences, College of Veterinary Medicine, North Carolina State University, Raleigh, NC USA; 4 Comparative Pain Research and Education Centre, College of Veterinary Medicine, North Carolina State University, Raleigh, NC USA; 5 Thurston Arthritis Center, UNC School of Medicine, Chapel Hill, NC USA; 6 Department of Anesthesiology, Center for Translational Pain Research, Duke University, Durham, NC USA

**Keywords:** Neuroscience, Medical research, Neurology

## Abstract

Maintaining an active lifestyle is considered a hallmark of successful aging. Physical activity significantly reduces the risk of cognitive decline and Alzheimer’s disease in humans. However, pain and lack of motivation are important barriers to exercise. Dogs are a remarkable model for translational studies in aging and cognition as they are prone to Canine Cognitive Dysfunction syndrome, which has many similarities with Alzheimer’s disease. According to owner reports, changes in activity levels are characteristic of this syndrome, with decreased daytime activity, but also excessive pacing, especially at sleep time. We used physical activity monitors to record the activity of 27 senior dogs and evaluated the association between activity level and age, fractional lifespan, cognitive status measured by an owner questionnaire and cognitive tests. We also assessed the relationship between activity and joint/spinal pain, and the off/on leash gait speed ratio (a potential marker of gait speed reserve and motivation). We found that activity patterns in dogs are associated with fractional lifespan and working memory. Additionally, dogs with higher on/off leash gait speed are more active in the afternoon of weekdays. These results encourage future studies evaluating how physical activity can improve or delay cognitive impairment in senior dogs.

## Introduction

Maintaining an active lifestyle has been linked to several health benefits and is considered a hallmark of successful aging^1^. Higher physical activity in older adults prevents diseases, reduces disability and improves quality of life^2,3^. Moreover, lower physical activity is one of the main domains considered in human frailty phenotype assessment^4^. Frailty is defined as a clinical state in late life characterized by an increased vulnerability for adverse health outcomes when exposed to a stressor^5^. Although benefits of physical activity in older people are well recognized, activity levels decline with age and only one third of older individuals follow exercise recommendations^6,7^. Kosteli et. al (2015) evaluated the determinants of lower activity in older adults and found that pain and lack of motivation are among the main barriers that older adults encounter when considering engaging in physical activity^8^. Mobility and cognitive health are interconnected; cognitive and motor impairment share several pathophysiological pathways^9^. In this regard, it has been demonstrated that higher physical activity significantly reduces the risk of cognitive decline and Alzheimer’s disease^10,11^.

Dogs, like people, suffer from age-related diseases and cognitive decline and are prone to dementia (i.e. canine cognitive dysfunction syndrome, CCDS)^12–14^. Additionally, older dogs are less active than younger dogs^15–17^. As in people, there is evidence suggesting that physical activity can be associated with cognitive performance in dogs. According to owner’s reports, one of the main behavioral changes that characterize CCDS is a change in activity levels; dogs usually show reduced activity during the day, but can also show excessive pacing, especially at night^18,19^. However, the relationship between physical and cognitive performance and the role of pain and motivation on activity have not yet been evaluated objectively in dogs.

In older people, self-selected gait speed, maximum gait speed and the gait speed reserve (ratio maximum gait speed/self-selected gait speed) have been shown to be reliable predictors of daily ambulatory activity^20^. This provides clinicians with a very practical insight into daily activity when performing several days of activity monitor recording is not feasible. In dogs, the potential relationship between gait speed and activity has not yet been studied. Since gait speed in dogs has been demonstrated to be influenced by body height^21^, and dogs have a clear body size diversity, the ratio of gait speed off leash toward a treat/gait speed on leash represents an interesting measure of gait speed reserve while normalizing for the effect of height.

Activity levels in dogs can be measured objectively using collar-mounted physical activity monitors (PAMs), and this approach is termed actigraphy. Most PAMs are accelerometers or inertial measurement units. Our group has already demonstrated that actigraphy can be used to identify rest and activity patterns in dogs and can detect differences in activity between different age groups and pain-treatment groups reliably using functional linear modeling (FLM)^17,22^. The aim of this work was to explore age-related changes in activity in senior and geriatric dogs by means of collar-mounted PAMs and to use FLM to determine if activity is associated with cognitive performance on owner questionnaire and cognitive testing. Furthermore, we evaluated the role of pain, an additional factor that may be associated with activity levels in aging dogs. Finally, the association between the off/on leash gait speed ratio and activity was also evaluated.

## Results

### Demographic information

We enrolled 27 dogs (12 spayed females and 15 neutered males), with a mean ± SD age of 12.9 ± 1.64 years and a fractional lifespan of 1.04 ± 0.12. As described in our previous work^23^, we calculated their fractional lifespan by dividing their age by their expected lifespan as proposed by Greer et al. (2007)^24^. Dogs belonged to 12 different breeds with 7 dogs (26%) being mixed breed. The most frequent breed was Labrador retriever (n = 5, 18.5%) followed by golden retriever, beagle and border collie (n = 2, 7.4% each). There was one dog of each of the following breeds: American Staffordshire terrier, Bernese Mountain dog, Cairn terrier, Pembroke Welsh corgi, dachshund, German shepherd, German short-haired pointer, Irish setter and Jack Russell terrier. According to the owners’ responses to the study questionnaire, the mean duration of active intentional exercise for these dogs was 22.8 ± 22.60 min a day, ranging from 0 to 82 min. Only three of the dogs were crated during any period of the day, two were crated for 8 h during the night (weekdays and weekends) and 1 from Monday to Friday, between 8 AM and 4 PM (while their owner was working). This dog was kept with another dog in a large run, and while the space was restricted, they had enough room to move around and interact with each other. A statistical evaluation of the impact of crating was not undertaken as there were so few dogs that were crated, but visual inspection of the data (Supplementary Fig. S1) suggested these dogs had very similar activity patterns to non-crated dogs.

### Cognitive evaluation

Every owner completed the CAnine DEmentia Scale (CADES) questionnaire^19^ and dogs scored a median of 10 (range 0–70). According to their score, ten dogs (37%) had their cognitive status classified as normal, with six (22%) dogs as mild, seven dogs (26%) as moderate and four dogs (15%) as severe cognitive impairment. Dogs performed a battery of cognitive tests; sustained gaze test, (to evaluate attention and executive function), cylinder tasks (inhibitory control and detour, to evaluate executive function and cognitive flexibility) and a working memory test. Every dog completed the first three tests, but the memory task was not performed in two of the dogs because they failed to pass warm-ups (required as a criterion for learning). These two dogs received a memory score of 0. The median sustained gaze time was 19.19 s (range 2.85–49.81), median memory threshold was 20 s (range 0–120), median percentage of correct trials in inhibitory control was 87.5 (range 0–100%) and in detour was 50 (range 0–87.5%).

### Evaluation of joint and spinal pain

Orthopedic and neurological examination was performed in all dogs^25,26^. Median joint pain score was 5 (range 0–17), and spinal pain was 2 (range 0–8), which generated a total median pain score of 6 (range 1–19). Severe pain was rarely found, one dog had severe pain in the left shoulder, one dog had severe thoracolumbar pain and two dogs had severe lumbosacral pain.

### Evaluation of gait speed

Gait speed on leash (letting the dog set the pace) and off leash (toward a treat) was measured in 26 (96%) of the 27 dogs. Median gait speed on leash was 0.77 m/s (range 0.41–1.30) and off leash speed toward a treat was 1.23 m/s (0.43–2.47). The median off/on leash gait speed ratio was 1.73 (range 0.77–3.33).

### Effect of aging on cognition, pain, and gait speed

Since aging is characterized by a constellation of physical, functional, and cognitive changes^27^, we assessed the correlation between the measured variables and fractional lifespan. The results of this analysis are shown in Table 1. We found that fractional lifespan was positively correlated with CADES score, and negatively correlated with sustained gaze and memory. There were also positive correlations between fractional lifespan and joint and spinal pain. Finally, fractional lifespan was not correlated with the performance at the cylinder tasks or with the ratio off/on leash gait speed.

**Table 1:** Multivariable non-parametric analysis showing the correlation between the variables analyzed in this study.

Variable	By variable	Spearman’s ρ	p value	Adj. p value
CADES	Fractional lifespan	0.673	< 0.001	< 0.001*
Sustained gaze	Fractional lifespan	−0.603	0.001	0.006*
Detour	Fractional lifespan	−0.376	0.053	0.103
Memory	Fractional lifespan	−0.566	0.003	0.016*
Inhibitory ctrl	Fractional lifespan	−0.410	0.034	0.098
Spinal pain	Fractional lifespan	0.475	0.012	0.048*
Joint pain	Fractional lifespan	0.556	0.003	0.015*
Off/on leash ratio	Fractional lifespan	−0.186	0.373	0.373

Significant differences (p < 0.05) are indicated by an asterisk.

### Actigraphy results

**Figure 1:**
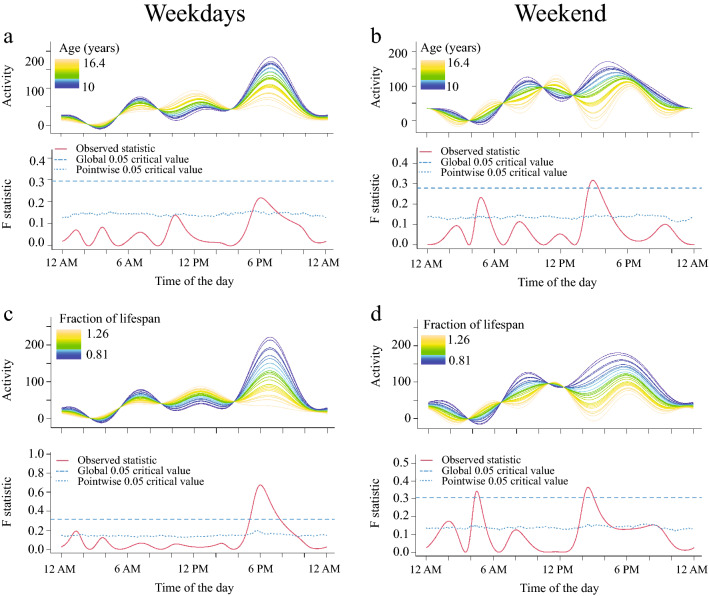
Relationship between age (**a,b**) and fraction of lifespan (**c,d**) on activity levels in senior dogs. For each figure, the upper graph illustrates activity over time for different levels of the variables analyzed and the lower graph indicates the level of significance of the differences. When the observed statistic (red line) is above the test of significance set at 0.05 (blue lines) it indicates significant differences between different levels of the variable. The dashed and the dotted lines indicate global and point-wise test of significance respectively. Older dogs showed lower activity levels between 5.00 and 8.00 PM on weekdays and between 2 to 5 PM on weekends. Also, during weekend nights, older dogs were more active around 5 AM.

**Figure 2:**
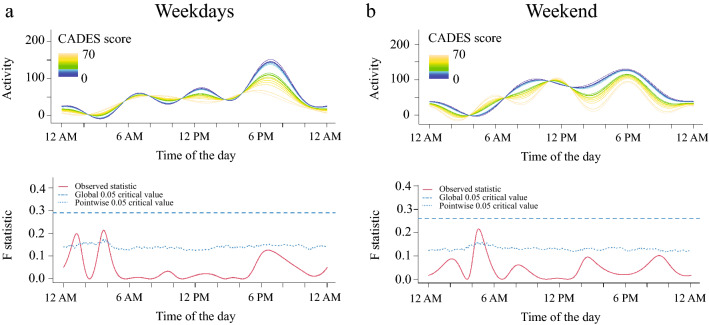
Relationship between CADES and activity levels in senior dogs on weekdays (**a**) and weekends (**b**). The upper graph illustrates activity over time for different levels of the variables analyzed and the lower graph indicates the level of significance of the differences. When the observed statistic (red line) is above the test of significance set at 0.05 (blue lines) it indicates significant differences between different levels of the variable. The dashed and the dotted lines indicate global and point-wise test of significance respectively. Dogs with higher CADES scores were more active between 3 to 5 AM during both weekdays (**a**) and weekends (**b**) and less active around 1 AM.

**Figure 3:**
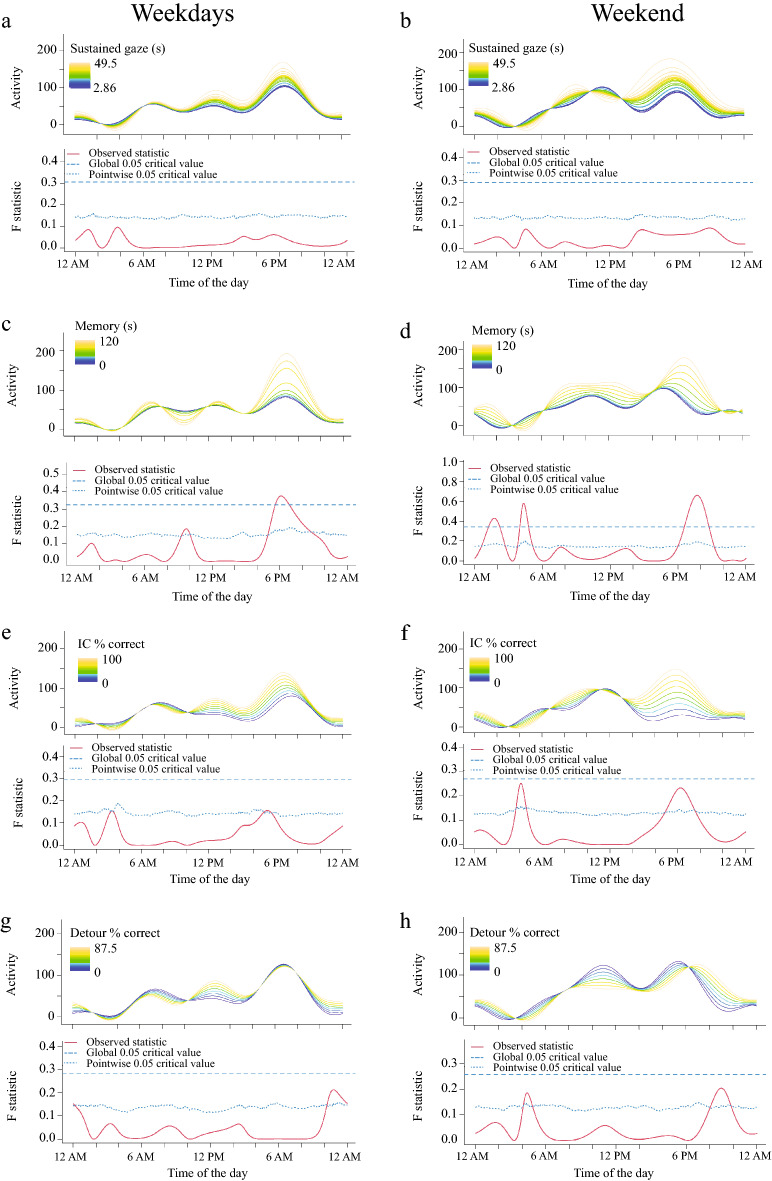
Relationship between performance on cognitive testing and activity levels in senior dogs on weekdays (**a,c,e,g**) and weekends (**b,d,f,h**). The upper graph illustrates activity over time for different levels of the variables analyzed and the lower graph indicates the level of significance of the differences. When the observed statistic (red line) is above the test of significance set at 0.05 (blue lines) it indicates significant differences between different levels of the variable. The dashed and the dotted lines indicate global and point-wise test of significance respectively. No relationship was observed with performance at sustained gaze (**a,b**). Dogs with higher scores in memory tasks were more active in the evening during weekdays (**c**) and weekend (d). On weekends they were also more active between 12 and 2.45 AM and less active between 3.45 and 5.30 AM. On weekdays, dogs with better performance also showed a reduction in activity levels between 9 and 10.15, but this did not surpass the global significance. Performance on the cylinder tasks, considered tests of executive function, was less clearly associated with activity levels (**e,g**). Differences were most notable at the weekend when dogs are more active, but non achieved global significance.

**Figure 4:**
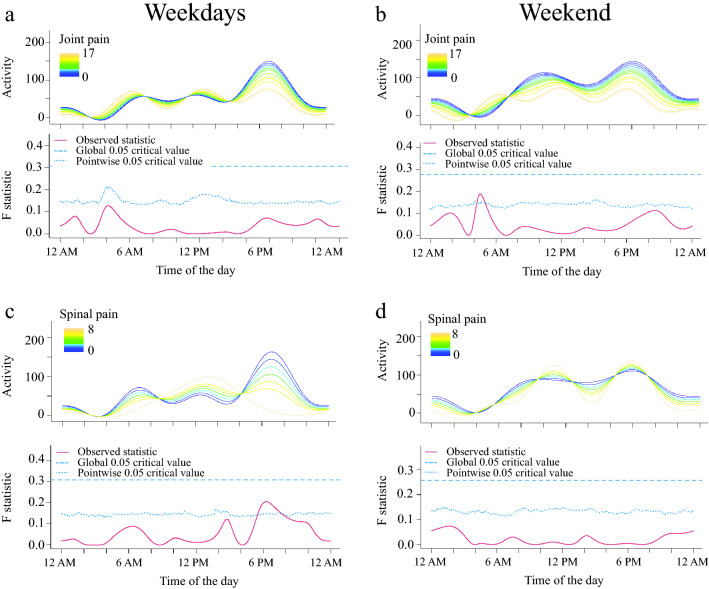
Relationship between joint (**a,b**) and spinal pain (**c,d**) on activity levels in senior dogs. The upper graph illustrates activity over time for different levels of the variables analyzed and the lower graph indicates the level of significance of the differences. When the observed statistic (red line) is above the test of significance set at 0.05 (blue lines) it indicates significant differences between different levels of the variable. The dashed and the dotted lines indicate global and point-wise test of significance respectively. Dogs with greater joint pain were more active between 4.15 and 5 AM during the weekend (**b**) but not during weekdays (**a**). Dogs with higher spinal pain were less active in the evening between 5.30 and 7.15 PM during weekdays (**c**) but not during the weekend (**d**).

**Figure 5:**
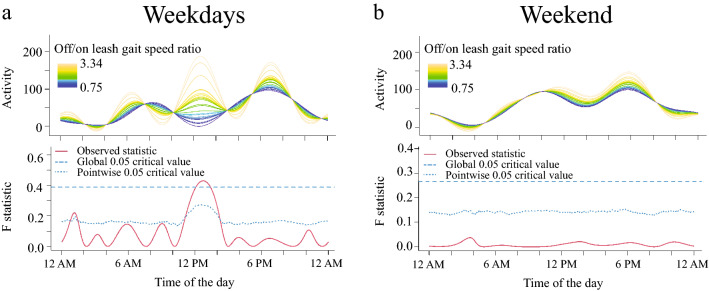
Relationship between off leash to on leash gait speed and activity levels in senior dogs during weekdays (**a**) and weekend (**b**). The upper graph illustrates activity over time for different levels of the variables analyzed and the lower graph indicates the level of significance of the differences. When the observed statistic (red line) is above the test of significance set at 0.05 (blue lines) it indicates significant differences between different levels of the variable. The dashed and the dotted lines indicate global and point-wise test of significance respectively. Dogs with higher levels of food-motivation were more active between 10.30 to 2.15 PM on weekdays.

Activity levels were measured over a 2-week period by means of a collar-mounted PAM (Actical monitor; Phillips Respironics). Results are shown in Figs. 1, 2, 3, 4 and 5. We found that activity levels in dogs fluctuated in a manner previously reported, with two clear peaks of activity in the morning (between 6 and 9 AM) and in the evenings (around 7 PM)^17,28^. Functional linear model analysis demonstrated an effect of age on weekday activity levels between 5 and 8 PM as it is shown in Fig. 1a. This age effect shows higher F statistic values and was only significant when assessed as fractional lifespan (Fig. 1c).

On weekends, FLM analyses showed that higher evening activity levels of both age (Fig. 1b) and fractional lifespan (Fig. 1d) were shifted towards an earlier time period, and that older dogs were more active than younger dogs in the early morning, reaching significance for fractional lifespan between 4.15 and 4.45 AM. As a result of these findings, fractional lifespan was used as the measure of age in multivariable analyses.

The FLM analysis showed that dogs with higher owner-assessed cognitive impairment (CADES score) were more active at late night/early morning during both weekdays (Fig. 2a) and weekends (Fig. 2b) and less active around 1 AM on weekdays. However, none of these findings reached global level of significance.

Activity levels were not associated with performance on the sustained gaze test or the cylinder tasks (Fig. 3a,b,e–h). By contrast, there was a significant association between performance on the memory task and activity. Dogs with higher memory scores were more active in the evening, during both weekdays (Fig. 3c) and weekends (Fig. 3d). During weekends, these dogs were also more active in the afternoon and dogs with worse performance showed higher levels of activity in the early morning.

We found that while dogs with higher appendicular joint pain scores tended to be more active in the early morning during weekends and dogs with higher spinal pain scores tended to be less active in the evening, neither joint nor spinal pain significantly influenced dogs’ activity (Fig. 4a–d). Finally, during weekdays, dogs with higher off/on leash gait speed ratio had higher activity in the early afternoon (Fig. 5a). No significant differences were observed between the off/on leash gait speed ratio and activity during weekends (Fig. 5b).

Since both fractional lifespan and memory showed significant associations with activity at approximately the same periods of time and memory decline is expected with aging in dogs^29^, we evaluated whether memory scores remained significantly associated with activity in a multivariable model including fractional lifespan as a covariate. Based on the FLM analyses, we selected the time periods between 5.45 and 6.45PM on weekdays and 6.30 and 8.45PM, 1.15 and 2.15AM, and 4 and 4.45AM on weekends and calculated the average activity/minute for each of these periods. During weekdays, the association between memory and activity in the evening (between 5:45 and 6:45 PM) did not remain significant (std β = −0.152, p = 0.461) and only fractional lifespan was correlated with activity (std β = −0.718, p = 0.002). The interaction between fractional lifespan and memory was also not significant and therefore dropped from the model. The model explained 41.1% of the variation in the activity at this period of time. By contrast, on weekends, the correlation between activity and memory performance did maintain significance for the evening period, between 6:30 and 8:45 PM (std β = 0.457, p = 0.039) while fractional lifespan showed no correlation with activity (std β = −0.220, p = 0.303). This model explained 37.6% of the variation in activity. The interaction of variables was not significant and therefore not included in the model. Neither memory nor fractional lifespan showed a significant association with activity for the period between 1:15 and 2:15AM (std β = 0.302, p = 0.207 and std β = −0.227, p = 0.339 respectively). In this analysis, the interaction between variables was also not significant. Finally, between 4 and 4:45 AM only the interaction between memory and fractional lifespan was correlated with activity (std β = 0.459, p = 0.033) but not the main effects of each variable (std β = 0.472, p = 0.062 for memory and std β = 0.058, p = 0.803). This model explained 26.5% of the variation in the activity at this period of time.

## Discussion

In this study we evaluated the activity patterns of a group of aging dogs with various degrees of cognitive impairment, joint and spinal pain, and with different gait speeds. As previously shown^29,30^, the degree of cognitive impairment in these dogs was correlated with their fractional lifespan. Additionally, both joint and spinal pain showed increased with fractional lifespan, which was expected since chronic pain conditions such as osteoarthritis are more likely to affect older dogs^31^. We measured gait speed using the off/on leash gait speed ratio, to control for conformational differences between dogs such as height, a parameter that has been shown to effect gait speed^21^. In this group of dogs, fractional lifespan was not associated with this ratio, while, in people, gait speed has been shown to decline with aging^32^. However, bigger differences are seen between young and older people, and frailty exacerbates these differences^9,32–35^ In our limited cohort of 27 dogs, all of which were elderly and able to meet the Neuro-aging Study inclusion criteria, which included independent ambulation and absence of serious comorbidities, we might not have had the statistical power needed to detect a relationship.

Use of the FLM analysis allowed us to identify activity signatures for all the aforementioned variables. We found that evening activity decreases with increased fractional lifespan in senior dogs. Age-related changes in activity levels were in agreement with other studies that found that younger dogs are more active than older dogs^17,28,36,37^. In fact, in companion, adult dogs, Brown et al. (2010) demonstrated a 4.2% decrease in total activity counts with every 1-year increase in age when dogs were trotting up and down stairs^38^. On the other hand, dogs with higher fractional lifespan were significantly more active at nighttime during weekends. These findings are in agreement with a previous study using radio-telemetry polysomnography that showed that older dogs have a tendency to sleep less during the night, especially between 4 and 5 AM^39^. The same study showed that dogs compensate for this sleep debt by sleeping more during the day. These age-related changes in sleep–wake cycle have been also demonstrated in humans^40^ and rodents^41,42^. The reasons why these changes were seen only during weekends need further research, nonetheless, several studies have shown that activity in pet dogs varies significantly between weekdays and weekends, with higher counts of activity recorded on weekends. These differences in activity reflect owners work patterns and our results suggest that examination of activity when owners are home and interacting more with their dogs might be the most useful period in which to examine activity levels in old dogs^17,28,43^.

The association between activity and fractional lifespan was more robust than chronological age. In dogs, body size is highly associated with lifespan; i.e. smaller breeds can have twice the expected lifespan than larger breeds^44^. Dogs are one of the species with the greatest diversity in body size^45^ and therefore in expected lifespan. This has led to questions of whether larger breeds develop age-related changes faster than smaller breeds, and whether this would occur across all health domains. According to the compression hypothesis, relative lifespan would drive age-related changes and so all sizes of dog would suffer from the same spectrum of age-related disease; this is in contrast with the truncation hypothesis in which age-related changes would be associated with chronological age^46^. This may be different across different health domains. While some studies have shown that larger dogs have an accelerated pace of physiological aging^47,48^, Watowich et al. (2020) showed evidence that age-related changes in cognition are independent of the breed lifespan and fit the truncation hypothesis^46^. The current study suggests that changes in activity in dogs may better fit the compression hypothesis. These results are in accordance with human studies, showing that mobility declines slowly across the lifespan fractions, and suffers a steeper decline in late stages^49^. Even though humans do not have the genetically influenced diversity in expected lifespan that dogs have, the critical age when this decline occurs is different for each individual person, and it occurs when the ability to compensate for the sum of impairments is depleted^49^.

We evaluated the association between activity levels and cognitive performance in dogs in 2 different manners. Firstly, we evaluated the owner-assessment of signs of dementia exhibited by their dogs at home using the CADES questionnaire, and secondly, we performed a battery of cognitive tests. The CADES questionnaire captures the classic signs of canine cognitive dysfunction syndrome (CCDS) as witnessed by the owners and our data showed no significant correlation between these scores and activity. These results might be due to the relatively low number of animals with severe cognitive impairment included in our study (n = 4). By looking at the activity curves, it is possible to observe that an activity signature was emerging with dogs with higher scores showing increased nighttime activity, however a larger number of dogs would need to be evaluated to investigate this further. Indeed, a recent article evaluated 11,574 dogs and showed a negative correlation between physical activity and severity of cognitive dysfunction symptoms by analyzing owner-reported questionnaires^50^. It is worthwhile noting that, in this article, activity levels were also measured by owner-reported questionnaires, i.e., no objective measure of activity was employed.

In order to examine specific cognitive domains, we evaluated dogs’ performance on cognitive tests that assess attention, memory, and executive function. Better performance in a task of working memory was associated with higher activity in the evening (the main peak of activity in dogs)^17,22^ and this correlation remained significant on weekends after including fractional lifespan in the model. Similarly, in older people, an association between higher levels of physical activity and better working memory (but not attention) has been demonstrated^51,52^. In laboratory dogs, Siwak et al. (2003)^36^ found that cognitively impaired dogs (evaluated by three neurophysiological tests) were more active than age-matched unimpaired dogs. However, this study differs from ours in two main aspects; first they used laboratory dogs in which activity is restrained by the laboratory settings and housing facilities, and second, the cognitive tests they employed were designed to evaluate learning abilities, not specifically working memory. While several studies in people have demonstrated that physical activity reduces the risk of developing mild cognitive impairment and Alzheimer’s disease by decreasing the amyloid-beta genesis and the production of reactive oxygen species involved in neurodegeneration and dementia^53,54^, patients with dementia are less likely to engage in physical activities, resulting in a vicious cycle^54,55^. It was notable that the dogs in our study did not typically exercise for long, with an average of only 22 min per day. Further studies evaluating whether promoting physical activity can improve or delay cognitive impairment in dogs are needed.

Performance in the memory task was also correlated with activity at night; dogs with worse performance were more active during the early morning and less active around 1 AM. When these relationships were evaluated in regression models, the interaction between memory and fractional lifespan showed a significant association with activity in the early morning. One of the main characteristics of CCDS is change in sleep–wake patterns, i.e., dogs show disruption of nighttime sleep, with some dogs even exhibiting aimless walking at night, and daytime sleepiness^19,56^. It has been demonstrated in several species, including dogs, that sleep is essential to memory consolidation and learning^57–59^. Additionally, in humans and rodents, sleep fragmentation and deprivation are associated with increased accumulation of amyloid beta in the brain (a key molecule in the pathophysiology of Alzheimer’s disease) and it has been shown that sleep contributes to its clearance^60,61^. Our data support the contention that CCDS-associated changes in the sleep–wake cycle and cognitive function (memory in particular) might be closely related.

Physical activity in dogs could be increased by treating comorbidities that reduce mobility. Muller et al. (2018) showed that higher degrees of impairment in osteoarthritis was associated with reduced activity^62^. Another study has shown that osteoarthritic untreated dogs were restless at night in comparison with dogs treated with an analgesic drug^22^. In people, it has been shown that pain is one of the main barriers that older adults experience to performing physical activity, but in this cohort of dogs, we did not find significant associations between joint or spinal pain and activity. We did note that dogs with higher joint pain scores tended to be more active in the early morning, as did dogs with higher CADES scores, highlighting the potential for interaction between these factors and activity. However, one limitation of this study is that dogs were not particularly painful; the median total pain score was 6, which is ~ 50% of total pain scores in dogs with painful osteoarthritis presenting for a clinical trial of an analgesic drug (unpublished data from a contemporaneous clinical study), and few had severe dementia. Therefore, to fully evaluate the role of pain in activity, and to examine the relationship between pain, cognition, and activity more fully, future studies should examine larger numbers of dogs and include age-matched dogs experiencing higher levels of pain.

### Off/on leash gait speed ratio

We found that the off/on leash gait speed ratio did play a role in activity during weekdays, around noon, the time between the usual times of peak activity in dogs. At this time there is a greater chance that owners might be out of the house working, and dogs might be left alone. Therefore, dogs who have a higher capacity to increase their gait speed in order to get a treat might be more willing to engage in physical activity even when they are without their owners. By measuring the ratio of off leash to on leash gait speed, we not only controlled for conformational differences^21^, but also likely captured the gait speed reserve and motivation (particularly food motivation) of the dogs. In people, the gait speed reserve (maximum gait speed/self-selected gait speed), self-selected and maximum gait speed (subjects are asked to walk as fast as possible) all correlate with activity levels and can be used as predictors of daily activity^20^. While there is no standardized measure of motivation in dogs, we also consider that this ratio might be influenced by motivation. Fukusawa et al. (2013) evaluated different reinforcements in dogs and demonstrated that dogs would be willing to run at a faster speed for a higher quality food-reward^63^. Motivation is considered a multidimensional construct that refers to the force with which animals (or people) choose particular actions at particular times or places. It results from the integration between the value of a reward and the effort required to obtain it^64^. In this study, a food reward was chosen because it is the most intrinsically rewarding incentive for most dogs, even more than praise or petting^65,66^. Of note, lack of motivation is also an important barrier to physical activity in older humans, and it is usually experienced by Alzheimer’s disease patients^67^. Further studies should aim to validate this off/on leash gait speed ratio as an actual measure of motivation in dogs.

### Limitations

This study had several limitations. Firstly, dogs were not living in a controlled environment and their activity was highly dependent on owners’ daily habits, available space in their houses, decisions of owners to keep dogs crated at specific times, etc. While the owners’ daily schedule will clearly influence the activity of their dogs, different study designs would be needed to address this issue, such as either simultaneously collecting activity data from the owner and dog, or requiring that owners follow a specific activity protocol. Secondly, we asked the owners about their dog’s typical exercise duration, whether (and when) dogs were crated and to record whenever the collar was removed, or the dog was doing an unusual activity, but we did not request owners to record their daily activity each day and so it was not possible to directly assess owner reported activities against activity data. Thirdly, of the dogs included in this study some had participated in a longitudinal study of neuro-aging for over a year and had performed cognitive testing every 6 months. It is possible that this repetition improved their cognitive testing scores, although our longitudinal data do not support this. Finally, the major limitation of this exploratory study was sample size. Future studies would benefit from analyzing a larger number of animals, especially including more dogs with severe cognitive dysfunction and more significant pain levels.

In conclusion, this study demonstrates that activity patterns in dogs are influenced by their fractional lifespan, and there are associations between activity levels and their working memory. Additionally, the off/on leash gait speed ratio could be a useful tool that provides an insight of the dogs’ levels of activity. These results encourage future studies evaluating how enhancing physical activity in senior dogs can improve or delay cognitive impairment.

## Methods

### Study population

Companion dogs from a range of different breeds and sizes, participating in a longitudinal study of neuro-aging at the North Carolina (NC) State University College of Veterinary Medicine, were included in this study. Dogs were recruited from January 2019 through June 2022 by advertising in social media, NC State email lists and NC State Clinical trials website. All owners reviewed an informed consent form and were given the opportunity to ask questions of the investigators prior to signing it. To be included, dogs’ fractional lifespan needed to be higher than 0.75 (i.e., to be older than 75% of their expected lifespan). The expected lifespan was calculated using formula developed by Greer et al. (2007) which takes height and weight into consideration since lifespan in dogs varies significantly with body size^24^. Fractional lifespan was calculated by dividing dog’s chronological age by their expected lifespan.

Dogs underwent physical, neurological, and orthopedic examinations and complete blood count, serum biochemistry and urinalysis were performed. Dogs were excluded if they were unable to walk independently and if they had comorbidities that would impede or interfere with cognitive testing such as blindness, or neurological and behavioral disorders that required the use of psychoactive drugs. Additionally, dogs that exhibit aggression toward the experimenter were not included. We did not exclude dogs with stable chronic diseases such as kidney disease or osteoarthritis.

All procedures were conducted with the approval of the North Carolina State University Institutional Animal Care and Use Committee (Protocol #21-303). All methods used were carried out in accordance with the PHS policy on the Humane Care and Use of Animals in Research. All the methods described are reported in accordance with the ARRIVE Guidelines.

In the longitudinal study of neuroaging dogs undergo three visits every 6 months. The data of their most recent set of three visits was used in this study. On the first visit clinical examination, bloodwork and urinalysis are performed. We also evaluate their mobility and gait speed on and off leash. A brief cognitive test is also performed on the first visit that serves as acclimation for the following visit. After the first visit owners were sent a set of questionnaires to complete, the ones used in this study were a questionnaire on dogs’ typical daily activities (including a question on whether (and when) dogs were crated, and one about the amount of time that dogs spent per day exercising or jogging), and the Canine Dementia Scale (CADES) questionnaire. On the second visit we performed a set of cognitive tests described below. Finally, on the third visit we performed additional evaluation (not relevant for this particular study), we mounted the PAM on the dog’s collar. The average time between each visit was 1 week.

### Cognitive evaluation

To determine the behavioral changes associated with CCDS, we asked the owners to complete the CADES questionnaire^19^. This questionnaire evaluates four domains of behavioral changes (spatial orientation, social interaction, sleep–wake cycles and house soiling) across seventeen questions. The total score ranges from 0 to 95 and allows classification of the dogs into four categories: normal (0–7), mild (8–23), moderate (24–44) or severe (45–85) cognitive dysfunction.

Additionally, we performed a set of cognitive tests in a designated cognitive testing room as detailed in Fefer et al. (2022)^29^. Every dog was tested in the morning, between 8 AM and 12 PM. During testing dogs had free access to fresh water and short breaks were provided when dogs seemed tired or to be losing interest (failing to make choices in the tasks). We evaluated attention (by means of sustained gaze test), working memory (determining for how long dogs were able to remember where a treat was hidden after increasing delays), executive function (by inhibitory-control cylinder task and the problem-solving detour cylinder task). The detailed methodology used for cognitive testing has been already described by our group and other authors^29,68–70^. In brief, sustained gaze consist of measuring the time dogs make eye contact with the experimenter when they hold a treat up near their eyes^70^. Working memory was evaluated by placing two red Solo® cups in a mat, and hiding a small food treat under one of them (in view of the dog). After putting the treat down, the experimenter would wait an incrementally increasing delay, and say “okay”. As soon as the handler hear the “okay” sign, they would release the dog so they could choose one of the two cups. Dogs received a food reward only if the correct cup was chosen. Nine different delays were tested, 3, 6, 10, 20, 40, 60, 80, 100 and 120 s. Time delay was only increased if dogs choose correctly 4 out of 6 trials. The longest delay with at least 4 correct trials out of 6 trials was recorded. Inhibitory control was evaluated by asking the dogs to retrieve a treat from inside of a horizontal transparent cylinder open on both ends. Food was placed on the center of the cylinder and dogs were allowed to approach the cylinder and retrieve the food. Trials were considered correct if the dog retrieved the food without touching the cylinder. This was repeated over 8 trials and the percentage of correct over total trials was calculated. For the detour task, this task increased its difficulty; the side most often used by dogs to retrieve the treat in the inhibitory control task was now blocked and dogs have to retrieve the treat using their non-preferred side.

### Pain evaluation

We scored pain by physical, orthopedic and neurological examination. The examination was done in a quiet room and treats were given frequently. Spinal pain was graded during palpation at the cervical, thoracolumbar, and lumbosacral regions from 0 to 3 as follows: 0: Does not notice; 1: Epaxial muscle tensing on palpation, 2: Muscle contraction and flinching, crouching, 3: Cries out, jumps away, tries to bite^25^. Scores at each region were summed to give a total score of 0–12. During orthopedic examination each appendicular joint was assessed, with all toes considered as one single site for each paw, so the joints evaluated were manus, carpus, elbow, shoulder, pes, hock, stifle, and hip. Joint pain was quantified by grading each joint during manipulation from 0 to 4 as follows: 0: Does not notice; 1: Orients to site, does not resist or mild resistance; 2: Orients to site, slight objection to manipulation; 3: Withdraws from manipulation, may vocalize, may turn to guard area and 4: Tries to escape/prevent manipulation, may bite or show aggression. Total joint pain was calculated as the sum of each joint score on a scale of 0–64^26^. As mentioned before, dogs that showed any signs of aggression towards the experimenter during a screening visit were not included in this study, and therefore signs of aggression during manipulation were likely related to pain and not to behavioral issues.

### Gait speed evaluation

Gait speed evaluation was performed in the gait lab of the CVM. This is a quiet room equipped with a flat indoor runway. To measure gait speed on leash the experimenter walked 5 m along the runway with the dog on leash allowing it to set its own pace (leash was always held loose) while recording the time. To determine speed off leash toward a treat, one experimenter held the dog at the beginning of the 5-m mark on the indoor course, while another experimenter showed the dog a treat saying the dog’s name and “Look”, moved to the end of the 5-m mark and called the dog. The dog was rewarded with the treat when they arrived at the experimenter. The time taken for the dog to move from the beginning to the end of the 5-m mark was recorded for both on- and off-leash testing and was divided by 5 to obtain the speed in meters per second. We repeated the testing in triplicate and calculated the average on and off leash speeds. The ratio of gait speed off leash to on leash speed (off/on leash gait speed ratio) was then calculated.

### Activity evaluation

Physical activity was measured over a period of ~ 2 weeks following the visit to the testing facility using a collar-mounted accelerometer. After clinical and cognitive evaluation, a PAM was mounted on dogs’ collars (Actical monitor; Phillips Respironics). The Actical monitor is a commercially available uniaxial accelerometer that has been shown to be a valid surrogate measure of distance moved in dogs^71^. The sampling rate was set to 30 Hz, and the epoch length was set to 60 s. We instructed the owners to keep the collar with the accelerometer on at all times for 2 weeks and to return the accelerometer after that period. We provided owners with a  log and asked them to record the dates and times of any unusual activity on their dog’s routine or unavoidable collar removal. Data were downloaded using Actical Software 3.11. The activity data underwent a quality control check, and data were excluded from days for which owners reported disruption of their routine, such as long hiking, parties at home, vacation days, etc. Additionally, daily graphs were inspected, and data were excluded from days in which there was complete lack of activity (counts of 0) for more than 3 h during daytime.

### Statistical analysis

Since both activity, and all the variables included in this study might be influenced by aging^23,29^, we performed a multivariate Spearman’s correlation analysis to determine the correlation between fractional lifespan and cognitive function (CADES score and performance in each cognitive test), joint and spinal pain and the off/on leash gait speed ratio. The Holm-Sidak familywise error correction was used to adjust the p value for multiple comparisons.

For the PAMs analysis, data from the first full week were used. Since it has been demonstrated that dogs’ activity is different on weekdays and weekends^17,28,72^, we analyzed data for the first 5 weekdays and for the first weekend separately. Using the raw per-minute activity counts over 5 weekdays, or 2 weekend days, we calculated values for the average per-minute activity for each dog for each minute of 24 h. Thus, each per-minute average reflected the mean of activity over a period of days for that particular timepoint in a 24-h day. Once averaged, data were processed using the RStudio 2021 (PBC, Boston, Massachusetts) package “Actigraphy” (version 1.4.0)^17,73,74^. This package uses functional linear modelling (FLM) analysis. This analysis has been specifically designed to examine actigraphy data by modeling the response variable as a function of time, providing much more information than classically used analyses that average the data over larger time periods. Functional linear modeling is a powerful tool when applied to high-frequency longitudinal data such as PAM output, but the approach does not allow for multivariate analysis—that is, only one factor can be evaluated at a time. However, FLM analysis can provide a framework for future work to understand the influences of multiple factors. Actigraphy package converts the raw activity data into a smoothed curve through a Fourier expansion model and performs a non-parametric permutation F-test to evaluate differences between individuals based on categorical or continuous variables. For this analysis we used 1000 permutations. P values were calculated by determining the proportion of permutation F values that were larger than the observed F statistic. Actigraphy package calculates the point-wise critical values (a curve with the F permutation proportion at each time point) and a global critical value (single number referring to the proportion of maximized F values from each permutation)^73^ to determine significance. Since the global test of significance is more robust, we consider differences between dogs to be significant only when the F values were higher than the global critical value^73,74^.

We assessed the effect of age and of fractional lifespan on activity patterns to evaluate whether age-related changes in activity were dependent on the proportion of lifespan (the life stage) or the chronological number of years alive (age in years)^46^. Furthermore, we evaluated how cognition (measured by CADES and cognitive testing) and pain (measured by clinical examination) influenced activity patterns. Finally, we evaluated whether the off/on leash gait speed ratio can provide an insight of daily activity in dogs. All these analyses were done for weekdays and weekends separately.

Since FLM analysis does not allow for multivariable analysis, for variables that significantly influenced activity at similar periods of time, we used a more standard approach^75^ and performed a multiple regression model analysis using the average activity per minute for those specific times to determine the contribution of each specific variable and their interaction to the activity levels.

## Supplementary Information

Supplementary Legends.

Supplementary Figure S1.

## Data Availability

The datasets generated during and/or analyzed during the current study are available from the corresponding author on reasonable request.
