# Supplementary material for: Activity patterns are associated with fractional lifespan, memory, and gait speed in aged dogs
Source: Sci Rep. 2023 Feb 14;13:2588. doi: 10.1038/s41598-023-29181-z (PMC9929073; doi:10.1038/s41598-023-29181-z)
Supplement: Supplementary file 1 — Supplementary Legends. [file 41598_2023_29181_MOESM1_ESM.docx]

**Supplementary Fig. S1 – Differences in activity between crated and non-crated dogs.**

Mean (solid line) and standard deviation (shadowed area) of activity of crated (n = 3) and non-crated dogs (n = 24) during weekdays and weekends. A statistical evaluation of the impact of crating was not undertaken as there were only three that were crated, but visual inspection of the data suggests that these two groups of dogs had very similar activity patterns.
