# Supplementary figures and images for: Activity patterns are associated with fractional lifespan, memory, and gait speed in aged dogs
Source: Sci Rep. 2023 Feb 14;13:2588. doi: 10.1038/s41598-023-29181-z (PMC9929073; doi:10.1038/s41598-023-29181-z)

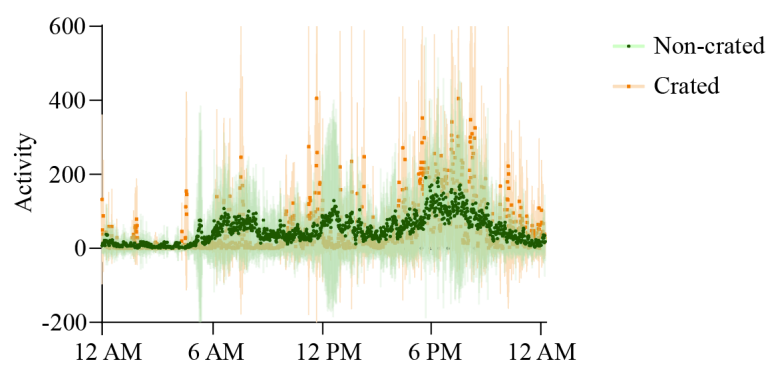

Supplement: Supplementary file 2 — Supplementary Figure S1. [file 41598_2023_29181_MOESM2_ESM.pdf]
